# Headache and MRI Changes after Endovascular Treatment of a Cerebral Aneurysm

**DOI:** 10.1155/2019/6917902

**Published:** 2019-12-20

**Authors:** Liv Jorunn Høllesli, Martin W. Kurz, Gry Inger N. Behzadi, Tore Solbakken, Svein Harald Mørkve, Kathinka D. Kurz

**Affiliations:** ^1^Department of Radiology, Stavanger University Hospital, 4068 Stavanger, Norway; ^2^Stavanger Medical Imaging Laboratory, Stavanger University Hospital, 4068 Stavanger, Norway; ^3^Department of Neurology, Stavanger University Hospital, 4068 Stavanger, Norway; ^4^Neuroscience Research Group, Stavanger University Hospital, 4068 Stavanger, Norway; ^5^Department of Clinical Medicine, University of Bergen, 5020 Bergen, Norway; ^6^Department of Neurosurgery, Haukeland University Hospital, 5021 Bergen, Norway; ^7^Department of Radiology, Haukeland University Hospital, 5021 Bergen, Norway; ^8^Department of Electrical Engineering and Computer Science, University of Stavanger, 4036 Stavanger, Norway

## Abstract

**Background:**

The main complications after endovascular therapy of intracranial aneurysms are aneurysm rupture and thromboembolic events. Yet, the widespread use of magnetic resonance imaging (MRI) in follow-up of these patients also demonstrates other, rarely known complications such as aseptic meningitis and foreign body reaction.

**Case Presentation:**

A small aneurysm in the right posterior communicating artery was treated with endovascular therapy in a 65 year old woman. Two weeks after successful interventional treatment, the patient developed a headache. On MRI performed five months after intervention, vasogenic edema was seen in the vascular territory of the right internal carotid artery. The edema and the symptoms diminished without specific treatment within a year.

**Interpretation:**

The clinical and radiological presentation of this case are suggestive of a foreign body reaction, a treatable condition that radiologists and clinicians should be aware of.

## 1. Introduction

Endovascular therapy (EVT) of aneurysms is a common treatment for cerebral aneurysms [1]. The complication rate of this procedure is relatively low, with the main complications being procedural aneurysmal perforations and thromboembolic events [2]. A rarely reported complication after EVT is delayed nonischemic cerebral enhancing (NICE) lesions [3]. These lesions have partly been attributed to foreign body emboli and subsequent granulomatous reaction [4]. There are some reports of foreign body reaction subsequent to EVT, yet only a few of them include imaging characteristics of these lesions and their development over time, especially in untreated cases showing the natural course. In this case we describe a probable foreign body reaction and its development over time, untreated as it was not recognized timely.

## 2. Case Report

A 65 year old female patient with a history of rare migraine attacks developed hyperacute severe global headache with accompanying symptoms of confusion and reduced short term memory. The headache was different from her previously known migraine, which had been asymptomatic for the last six years. The symptoms dissolved completely within 90 minutes. 2.5 weeks later she consulted a doctor, and was admitted to the hospital.

On neurological examination there were no focal neurological deficits. There were no paresis or numbness in the limbs, and cranial nerve examination and reflexes were normal. Physical examination was also completely normal.

On admission an unenhanced Computer tomography (CT) of the head was performed. There were no signs of haemorrhage or other pathology. CT angiography of the intracranial arteries revealed a 2-3 mm aneurysm in the right posterior communicating artery. There was an anatomical variant with fetal origin of the right posterior communicating artery and a hypoplastic P1-segment of the right posterior cerebral artery. This aneurysm was assumed to have caused “warning leak,” indicating an unstable aneurysm in need of treatment.

The aneurysm was successfully treated with carotid artery stenting and aneurysm coiling. Postinterventionally, the patient was clinically asymptomatic without any neurological deficits. The patient was treated with Acetylsalicylic acid and Ticagrelor, as Clopidogrel did not show adequate effect by platelet function tests. The patient was discharged to her home with a planned follow-up for Magnetic resonance imaging (MRI) of the brain three and twelve months postinterventionally.

One month after EVT, the patient was rehospitalized due to two weeks of right sided headache and a transient episode with flickering in the left visual field. A CT of the head and a CT angiography were performed and revealed small areas of subacute infarction in the right insula, interpreted to be due to a procedure-related thromboembolic event. There was no bleeding, and cerebral arteries were open. Neurological examination was normal. Except for soreness in the neck muscles, the medical examination was normal. The patient's symptoms were thought to be caused by tension headache, and the patient was discharged the day after.

One week after hospital dismissal, the patient was again rehospitalized due to persisting headache and a transient episode of anomic aphasia. Additional to a new CT of the head and CT angiography, a CT perfusion of the head and a brain MRI were performed. The examinations revealed no new findings. The patient had a slight persistent aphasia at admission which completely resolved within an hour. Blood samples were normal. The headache was still thought to be caused by tension headache, and the patient was dismissed after four days.

**Figure 1:**
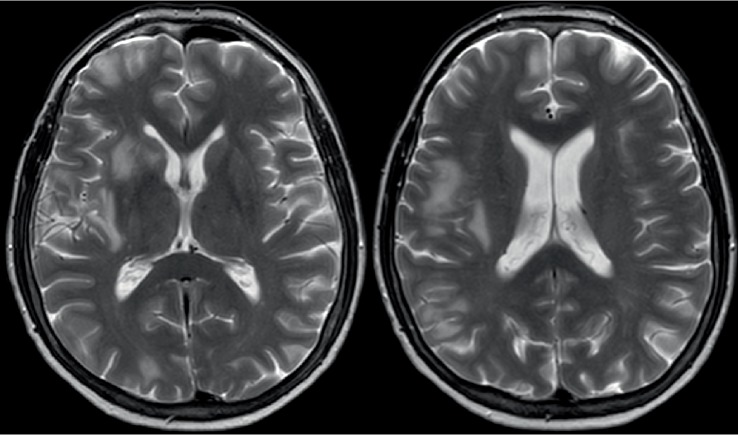
Transversal T2-weighted MR images five months after endovascular treatment show high-signal white matter changes with slight mass effect in the vascular territory of the right internal carotid artery.

Five months after EVT, a follow-up brain MRI was performed. On T2-weighted images, new hyperintense white matter signal changes consistent with vasogenic edema in the vascular territory of the right internal carotid artery were seen (Figure 1). There were approximately 10 lesions located mainly subcortical in the vascular territory of the right internal carotid artery. The lesions had slight mass effect. There was no restricted diffusion. Contrast enhanced MRI was not performed.

**Figure 2:**
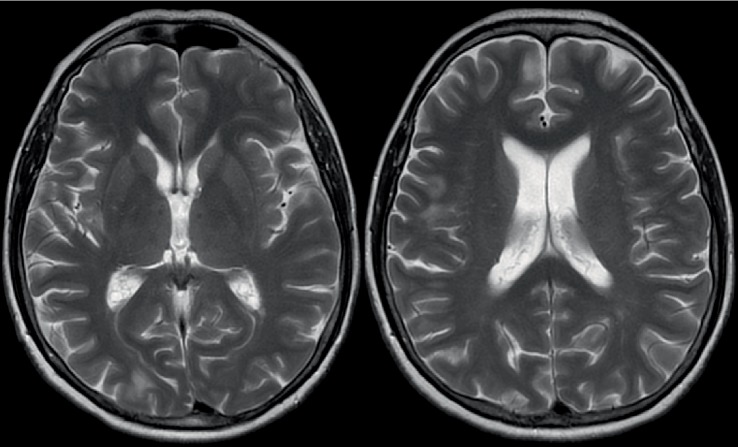
Transversal T2-weighted MR images one year after endovascular treatment revealed only subtle remaining signal changes.

One year after EVT, a new follow-up MRI was performed, showing only small remaining areas of signal changes (Figure 2). The patient's neurological examination was still normal, and the headaches decayed about six months after EVT without specific treatment. The clinical and radiological presentation were interpreted to be suggestive of a foreign body reaction after neurointerventional treatment.

## 3. Discussion

Foreign body reaction is a rare complication after EVT of intracranial aneurysms [4–8]. Typically punctuate, nodular or annular foci of leptomengial, cortical, and subcortical enhancement and vasogenic T2-hyperintense peri-lesional edema within the territory of EVT treatment are seen. In our patient several mainly subcortical T2-hypertintense lesions consistent with vasogenic edema were found in the vascular territory of the EVT treatment.

The incidence of foreign body reaction after intracranial aneurysm EVT is assumed to be around 0.5% [9, 10]. Yet, probably the incidence is higher as asymptomatic patients may be missed if they are not followed up with routine MRI. Patients with foreign body reaction after EVT are reported to have various symptoms including seizure, headache, motor deficit, involuntary movements, spasms, visual disturbances, central facial nerve palsy, and speech disturbances [9]. Our patient had persisting headache developing a couple of weeks after EVT. She also had a transient episode with flickering in the left visual field and a transient episode of anomic aphasia.

There is still some uncertainty in the mechanisms causing the signal changes on MRI. Endovascular devices have a surface covered with hydrophilic polymers to minimize endothelial friction and trauma during procedures. Friction between devices during endovascular treatment may lead to small emboli of hydrophilic micropolymers, which in turn is thought to cause a foreign body reaction in the tissue [9].

Histopathologically, various patterns are found, where inflammatory, embolic, and arteriopathic are among the most important. A paper from 2016 systematically reviewed clinical and diagnostic features in 32 patients in whom intracranial polymer reactions were documented following intravascular interventions [11]. The following types of histopathological patterns were most common: Perianeurysmal inflammatory changes manifested as aneurysm wall thickening and perianeurysmal parenchymal edema. Inflammatory changes centered around the polymer emboli was also found in the brain parenchyma downstream from sites of intervention with mostly granulomas but also sterile abscesses in some patients. Polymer embolization was also shown to cause artery occlusion, usually of small arteries, and parenchymal infarcts.

Typical MRI features are peri-aneurysmal brain edema, and there may also be an enhancing mass centered in the periphery of the wrapped aneurysm. Additionally there may be areas of high T2 signal in the vascular territory of the EVT treated artery, with central diffusion restriction and a thin rim enhancement [12, 13]. In our patient, the MRI showed findings consistent with vasogenic edema in the vascular territory of the EVT treated artery.

Headache has also been described as a complication after internal carotid artery stenting. This could contribute to our patient's headache. A case report from 2011 describes an increasing expansion with mass effect in the right frontal lobe distal to a coiled internal carotid artery bifurcation aneurysm. This expansion increased over several years. It was surgically resected, and described as a reactive, noninfectious process most likely resulting from the coils acting as a foreign body [6]. However, our patient had several brain lesions with only slight mass effect, normalizing within a year.

We do not have the diagnosis histologically verified, but the clinical presentation and MRI findings are consistent with the diagnosis of a foreign body reaction after neurointerventional treatment [7, 9].

Being aware of this potential complication after endovascular treatment is important, and in some cases treatment with anti-inflammatory medication is indicated. Shapiro et al. reported complete clinical resolution and absence of perilesional edema on follow-up imaging after treatment with a two-month course of dexamethasone. Yet, optimal dose and duration of this therapy is not established and needs to be further investigated [10].
